# Nicotinic receptor modulation to treat alcohol and drug dependence

**DOI:** 10.3389/fnins.2014.00426

**Published:** 2015-01-15

**Authors:** Shafiqur Rahman, Eric A. Engleman, Richard L. Bell

**Affiliations:** ^1^Department of Pharmaceutical Sciences, College of Pharmacy, South Dakota State UniversityBrookings, SD, USA; ^2^Department of Psychiatry, Institute of Psychiatric Research, Indiana University School of MedicineIndianapolis, IN, USA

**Keywords:** nicotinic receptor, alcohol dependence, nicotine addiction, drug addiction, CNS disorders, drug development, animal models

## Abstract

Alcohol and drug dependence are serious public health problems worldwide. The prevalence of alcohol and drug dependence in the United States and other parts of the world is significant. Given the limitations in the efficacy of current pharmacotherapies to treat these disorders, research in developing alternative pharmacotherapies continues. Preclinical and clinical evidence thus far has indicated that brain nicotinic acetylcholine receptors (nAChRs) are important pharmacological targets for the development of medications to treat alcohol and drug dependence. The nAChRs are a super family of ligand gated ion channels, and are expressed throughout the brain with twelve neuronal nAChR subunits (α2–α10 and β2–β4) identified. Here, we review preclinical and clinical evidence involving a number of nAChR ligands that target different nAChR subtypes in alcohol and nicotine addiction. The important ligands include cytisine, lobeline, mecamylamine, varenicline, sazetidine A and others that target α4β2^*^ nAChR subtypes as small molecule modulators of the brain nicotinic cholinergic system are also discussed. Taken together, both preclinical and clinical data exist that support nAChR–based ligands as promising therapeutic agents for the treatment of alcohol and drug dependence.

## Overview-alcohol and drug dependence: nicotinic receptors

Alcohol and drug dependence are chronic neuropsychiatric and relapsing disorders and represent a significant public health problem worldwide (Koob and Volkow, 2010; Volkow and Baler, 2014; Wise and Koob, 2014). Furthermore, fetal alcohol syndrome caused by alcohol exposure *in utero* is the number one preventable cause of cognitive and attentional deficits (Niccols, 2007; Leibson et al., 2014). The prevalence of alcohol abuse and dependence in the United States is about 8.5% with an estimated annual cost of 185 billion dollars (Litten et al., 2012; Davies et al., 2013). In addition, due to limited efficacy with existing US Food and Drug Administration (FDA)-approved medications for alcohol dependence, such as naltrexone and acamprosate, and high relapse rates, there is a need for alternative brain targets (Volkow and Skolnick, 2012). These brain targets will open new avenues for better treatment strategies targeting alcohol or drug dependence by interrupting the dependence and relapse cycle. The current review aims to cover the currently available pharmacological and therapeutic approaches involving nicotinic acetylcholine receptors (nAChRs) relevant to alcohol and drug dependence. In addition, this review will discuss the current status, putative mechanisms of action, and future directions for research into cholinergic treatments targeting alcohol and drug dependence. We believe molecular targets within the nAChR system offer great potential for developing pharmacotherapies to treat alcohol dependence and other addictive disorders. Moreover, neural circuits regulating cognitive activities such as decision-making and associated behaviors are negatively impacted by chronic alcohol or nicotine exposure (Clark and Robbins, 2002; Noël et al., 2013). Therefore, potential use of nAChR-based ligands and their ability to reverse some of these negative effects could improve impaired cognitive function of alcoholics and addicts and enhance the effectiveness of cognitive and behavioral therapies (Chatterjee and Bartlett, 2010).

A role for brain, ligand-gated, membrane bound ion channel-associated nAChRs in alcohol and drug dependence has been well-documented (see Feduccia et al., 2012; Rahman and Prendergast, 2012; Rahman, 2013; Hendrickson et al., 2013). The nAChRs are ionotropic or ligand-gated ion channels which belong to a superfamily of homologous receptors including glycine, serotonin type 3 (5-HT3), and γ-amino butyric acid (GABA) receptors (Dani and Bertrand, 2007; Hurst et al., 2013). The nAChRs in the mammalian central nervous system regulate processes such as neurotransmitter release, cell excitability, and neuronal integration and influence physiological functions, including arousal, sleep, mood, pain, and cognition (Klink et al., 2001; Hogg et al., 2003; Albuquerque et al., 2009; Gotti et al., 2009). The nAChR ion channel is formed by five membrane-spanning subunits which allow passage of cations like Na^+^ and Ca^++^. Each subunit has a long extracellular hydrophilic N-terminus containing the ligand binding domain, four hydrophobic transmembrane domains (TM1–TM4), and a short carboxy terminus facing the extracellular surface (Champtiaux et al., 2003; Albuquerque et al., 2009). The TM2 domain forms the inner lining of the cation channel and the anionic amino acids in this domain regulate ion conductance through the pore. The brain nAChR subunits are classified as alpha (α2–α10) or beta (β2–β4), according to the protein sequence and presence in the N-terminal domain of the α-subunits of two adjacent cysteines at positions 192 and 193, which are thought to participate in the ligand binding site. While the residues in the α-subunit form the primary face of the agonist binding site and determine the affinity for the ligand, the β-subunit forms the complementary face of the binding site and contributes to ligand selectivity (Gotti et al., 2009). Heteromeric receptors are assembled from both alpha (α2–α6) and beta subunits, while homomeric receptors are formed by alpha subunits only. Thus, heteromeric nAChRs contain two ligand binding sites (at the interface of α and β subunits), whereas homomeric nAChRs contain five ligand binding sites.

The nAChRs with identical subunit composition may differ in the stoichiometry of subunits, thus contributing to the variable channel kinetics, agonist binding, and pharmacological heterogeneity of nAChRs (Champtiaux et al., 2003; Moroni and Bermudez, 2006; Millar and Gotti, 2009). For example, (α4)_2_(β)_3_ nAChR subtypes are more sensitive (show higher affinity) to agonists in comparison to low affinity (α4)_3_(β)_2_ subtypes, indicating that changes in nAChR stoichiometry can elicit different physiological and pharmacological responses (Nelson et al., 2003). More than 90% of the receptor subtypes in the rodent brain are α4β2 nAChRs (Flores et al., 1992; Gotti et al., 2007; Albuquerque et al., 2009). However, the β2 subunit is less ubiquitous in the human brain (Paterson and Nordberg, 2000). The α4β2 nAChRs are widely distributed in various neuroanatomical regions, including the mesocorticolimbic dopamine system (Perry et al., 2002; Zoli et al., 2002; Gotti et al., 2007, 2009, see Table 1). The α7 nAChRs are also highly expressed in the brain and predominantly located in the hippocampus, cortex, and subcortical limbic regions (Gotti et al., 2007, 2009, see Table 1). Previous studies suggest that nAChRs are expressed at the synapse, cell body, and axons (Livingstone and Wonnacott, 2009). Presynaptic nAChRs are involved in regulating the release of ACh (Wilkie et al., 1993), NE (Clarke and Reuben, 1996), dopamine (Grady et al., 1992), glutamate (Alkondon et al., 1997), and GABA (Yang et al., 1996). Evidence indicates that dopamine release is modulated by α4β2^*^, α3β2^*^, and α6^*^ nAChRs (^*^indicates possible involvement of other receptor subunits) in nigrostriatal terminals (Le Novere et al., 1996; Luo et al., 1998; Wonnacott et al., 2000; Salminen et al., 2004). Glutamate release is regulated by presynaptic α7 nAChRs (Mansvelder et al., 2002). Similar to other ligand-gated ion channels, nAChRs modulate the flow of ions across the cell membrane under the influence of an extracellular signaling molecule. A net influx of cations (Na^+^, Ca^++^) through the ion channel depolarizes the cell membrane and increases neuronal excitability. The Ca^++^ entry through some nAChRs exerts additional effects on intracellular signaling cascades. ACh, the endogenous ligand of nAChRs, is released from the presynaptic cholinergic axon terminals and binds to the extracellular ligand binding domain of the receptor. Binding of ACh or exogenous ligands to the orthosteric site influences transition rates between three distinct functional states of nAChRs: the resting, open, and desensitized states. The rate constants between the functional states are dependent on the specific combination of subunits and the chemical characteristics of the ligand that is bound. Prolonged exposure to small doses of nicotine rapidly activates nAChRs initially, which is followed by desensitization of various nAChR subtypes (Quick and Lester, 2002). Heteromeric subtypes such as α4β2^*^ or α6β2^*^ slowly desensitize in an activity-dependent manner when exposed to low concentrations of nicotine, whereas homomeric subtypes such as α7^*^ nAChRs are much less susceptible to desensitization (Wooltorton et al., 2003) to low concentration of nicotine. The nAChR subtypes are stimulated or blocked by a number of agonists or antagonists (Gotti et al., 2007, 2009, see Table 1). Furthermore, brain nAChRs can be desensitized by continuous or repeated exposure to an agonist (e.g., nicotine) that results in progressive decreases in response to the drug. However, antagonism produced by specific ligand binding to the nAChR is somewhat different pharmacologically from these desensitized states (Buccafusco et al., 2009).

**Table 1:** **Localization of brain nAChR subtypes (heteromeric^1^ and homomeric^2^)**.

Prefrontal cortex	α4β2^*^^a^^,^^b^, α7^c^^,^^d^
Ventral tegmental area	α4β2^*^^a^^,^^b^, α6β2^*^ α3β4^*^, α7^c^^,^^d^
Nucleus accumbens	α4β2^*^^a^^,^^b^, α6β2^*^ α3β4^*^, α6α4β2^*^
Hippocampus	α7^c^^,^^d^, α4α5β2, α4β2^a^^,^^b^
Amygdala	α4β2^*^^a^^,^^b^, α7^c^^,^^d^

* *Indicates other α or β subunits such as α3, α5 or α6 and β3 or β4*.

a ACh and nicotine are agonists of α4β2^*^ subtype^3^.

b Dihydro-β-erythroidine and mecamylamine are antagonist of α4β2^*^ subtype^4^.

c *ACh, nicotine and choline are agonists of α7 subtype^5^*.

d *α-bungarotoxin, mecamylamine, methylylcoconitine and conotoxin are antagonist of α7 subtype^6^*.

1 *Flores et al., 1992; Zoli et al., 1998; Paterson and Nordberg, 2000; Perry et al., 2002; Gotti et al., 2007, 2009; Albuquerque et al., 2009*.

2 *Gotti et al., 2007, 2009*.

3 *Quick and Lester, 2002; Champtiaux et al., 2003; Nelson et al., 2003; Moroni and Bermudez, 2006; Gotti et al., 2007, 2009*.

4 *Larsson and Engel, 2004; Gotti et al., 2007, 2009*.

5 *Wooltorton et al., 2003; Gotti et al., 2007, 2009*.

6 *Gotti et al., 2007, 2009; Kamens et al., 2010; Crooks et al., 2014*.

Recent work with nAChR subtype knockout (KO) mice have provided important information on both brain nAChR function and their mediation of addiction related behavior (Fowler et al., 2008; Mineur and Picciotto, 2008; Changeux, 2010). For example, early research showed that mice lacking the β2 subunit do not display several nicotine-associated responses, including nicotine-induced DA release in the dorsal and ventral striatum as well as, nicotine-elicited increases in the firing rate of associated DA neurons (Picciotto et al., 1995, 1998). The lack of nicotine's effect on the mesolimbic DA systems in β2 subtype nAChR KO mice is consistent with the absence of nicotine self-administration by these animals (Picciotto et al., 1998). The α4 subunit requires the β2 subunit for assembly in the majority of heteromeric nAChRs in the brain, these and other studies using genetically modified mice suggest that α4/β2^*^ nAChRs are critical for nicotine-related reward behaviors (Ross et al., 2000; Tapper et al., 2004). Despite the distribution of the α7 subunit in the brain, in particular its presence in the mesocorticolimbic system, studies in α7 KO mice are not definitive about a role for the α7 subunit in nicotine reward and conditioning (Mineur and Picciotto, 2008). However, α7^*^ nAChRs are important for long-term potentiation, neuroplasticity associated with learning and memory, in the mesolimbic reward pathway (Mineur and Picciotto, 2008). KO mouse studies targeting the α6 subunit indicate that α6 partners with β2 nAChRs and may play an important role in nicotine addiction related behavior (Champtiaux et al., 2002). Recently, studies with transgenic over expression of the α5, α3, and β4 receptor subunit genes indicate these subunits have a potential, but complex, role in the modulation of nicotine related behaviors (Gallego et al., 2012).

Similar to research on the involvement of AChR subunits in nicotine-induced behaviors, a number of genetic studies have been conducted to identify the role of nAChR subtypes in alcohol drinking behavior. For example, acute alcohol drinking behavior is reduced in α4 KO mice compared to wild type (WT) indicating a role for the nAChR α4^*^subunit in alcohol abuse (Hendrickson et al., 2010, 2013). Similarly, alcohol-related behaviors and alcohol-induced midbrain dopaminergic function is decreased in α4 KO mice (Liu et al., 2013). On the other hand, β2 KO mice behave similarly to WT type mice in alcohol drinking behaviors (Kamens et al., 2010). In addition, α6 KO and β3 KO mice also display alcohol drinking behavior that is similar to WT mice in a two-bottle alcohol drinking paradigm (Kamens et al., 2010). Moreover, α7 KO and WT mice consume similar amounts of alcohol, although there was a potential gender effect regarding α7 nAChRs effects on ethanol consumption (Kamens et al., 2010). And, again, α5 KO mice do not differ in acute ethanol consumption compared to WT mice (Santos et al., 2012). Like nicotine-related behavior (see above), studies with transgenic over expression of the α5, α3, and β4 receptor subunit genes indicate these subunits have a complex role in the modulation of alcohol related behaviors (Gallego et al., 2012). Together, these data indicate that nAChRs containing α5, α6, β2, or β3 subunits may not be critical in alcohol drinking behaviors. Overall, the evidence indicates that α4 receptors in the midbrain may be associated with alcohol related behavior. Taken together, brain nAChRs represent a diverse class of receptor subtypes which are involved in a number of neurobiological functions and are associated with neurological and psychiatric disorders, including nicotine and alcohol dependence.

## Nicotinic receptors: targets to treat alcohol dependence

As with the treatment of alcohol dependence, the existing FDA-approved medications for nicotine dependence such as bupropion and varenicline, have had limited efficacy with continued significant relapse rates (Volkow and Skolnick, 2012). These brain targets will open new avenues for better treatment strategies targeting alcohol or drug dependence by interrupting the dependence and relapse cycle. Research indicates that brain nAChR subtypes are important mediators of the rewarding effects of alcohol (ethanol) and drugs of abuse (Blomqvist et al., 1993; Ericson et al., 1998; Lê et al., 2000; Soderpalm et al., 2000; Chi and de Wit, 2003; Young et al., 2005; Reus et al., 2007; Steensland et al., 2007; Bell et al., 2009; Liu et al., 2013). It is widely known that systemic or local administration of mecamylamine, a non-selective nAChR antagonist reduces ethanol drinking in a number of animal models (Ericson et al., 1998; Lê et al., 2000; Soderpalm et al., 2000; Steensland et al., 2007). Also, it has been proposed that nAChRs in the VTA regulate ethanol consumption and associated mesocorticolimbic neurochemical effects (e.g., dopamine release) in various animal models (Ericson et al., 1998; Chi and de Wit, 2003). However, mecamylamine either reduces or fails to decrease ethanol drinking behavior in humans (Blomqvist et al., 1996, 2002; Young et al., 2005), indicating mixed efficacy for treating ethanol dependence through nAChR blockade. Understandably, these mixed results have limited mecamylamine's clinical utility for ethanol drinking cessation. On the other hand, a selective α4β2 antagonist, dihydro-β-erythroidine failed to suppress ethanol consumption, thus suggesting a role for α6β2^*^ but not the α4β2^*^ subtypes in alcohol reinforcement (Larsson et al., 2002; Larsson and Engel, 2004). Similarly, the α7 nAChR antagonist methyllycaconitine was ineffective in reducing ethanol intake in an animal model of excessive ethanol drinking (Kamens et al., 2010). Varenicline, a partial α4β2^*^ nAChR agonist and FDA-approved medication for smoking cessation (Reus et al., 2007), was found to reduce alcohol drinking in both animal models and humans (Steensland et al., 2007; McKee et al., 2009, 2013; Hendrickson et al., 2010; Kamens et al., 2010; Bito-Onon et al., 2011; Chatterjee et al., 2011; Sajja and Rahman, 2011, 2013a; Mitchell et al., 2012; Litten et al., 2013; Sotomayor-Zarate et al., 2013; Kaminski and Weerts, 2014). The drug was developed as a potent high-affinity partial agonist at α4β2^*^ nAChRs (Reus et al., 2007), but also targets other nAChR subtypes as well. Therefore, the role of specific nAChR subtypes needs further investigation. Additional nAChR ligands such as cytisine, a partial agonist at α4β2^*^ and lobeline, a non-selective antagonist were found to reduce alcohol consumption and nicotine-induced alcohol drinking (Bell et al., 2009; Hendrickson et al., 2009; Chatterjee et al., 2011; Sajja and Rahman, 2011, 2012, 2013a).

These nAChR ligands also altered alcohol-induced increases in mesolimbic tissue DA levels (Sajja et al., 2010) in mice, confirming the important role of nAChRs in alcohol drinking and suggesting their involvement in alcohol dependence. Moreover, cytisine and lobeline were found to decrease alcohol self-administration in high alcohol drinking rats (Bell et al., 2009), a genetic animal model for alcohol abuse and dependence (Bell et al., 2012), and mice (Sajja and Rahman, 2011), suggesting that lobeline and cytisine are strong candidates for treating alcohol dependence. Sazetidine-A, a novel compound that selectively desensitizes α4β2 nAChRs, with partial agonistic activity (Xiao et al., 2006; Rezvani et al., 2013), was shown to reduce alcohol drinking in alcohol-preferring rats (Xiao et al., 2006; Rezvani et al., 2010). The evidence suggests that the desensitizing effects of sazetidine on α4β2 nAChR subtypes may account for these reductions in alcohol self-administration. Overall, sazetidine-A may have potential for the management of alcohol dependence by targeting brain nAChR-associated mechansims.

The existing animal and human studies suggest that alcohol-induced activation of the mesolimbic DA system involves brain nAChR stimulation. The rewarding effects of alcohol are dependent on the activation of the nAChRs in the mesolimbic DA system (Rollema et al., 2007). Overall, it is clear that brain nAChRs have emerged as critical targets for the reinforcing actions and DA activating effects of alcohol. Thus, ligands or compounds targeting nAChRs, other than those selective for α4β2 nAChRs have potential for treating alcohol dependence in humans. For example, CP-601932 and PF-4575180, partial agonists at α3β4^*^ nAChR were found to reduce alcohol consumption and preference in rats, confirming a role for additional nAChR subtypes in alcohol dependence (Chatterjee et al., 2011). Overall, nAChR partial agonists, antagonists or other ligands (see Table 2) target several nAChRs, such as α4β2^*^ and/or α3β4^*^ in order to modulate alcohol self-administration, underscoring the need to conduct more subunit-specific nAChR research regarding alcohol abuse and dependence. While nAChR ligands or partial agonists show great promise in reducing alcohol self-administration, evidence indicates that these ligands also decrease the alcohol deprivation effect, a validated animal model of relapse behavior (McKinzie et al., 1998; Spanagel and Hölter, 1999; Rodd et al., 2004; Melendez et al., 2006; Sparta et al., 2009; Bell et al., 2012). Emerging preclinical studies suggest that nicotine exposure re-instates alcohol seeking behaviors in rodents following extinction of alcohol reinforcement (Lê et al., 2003; Hauser et al., 2012). Furthermore, nAChRs were found to regulate deprivation-induced re-exposure of alcohol seeking in long-term alcohol exposed animals (Kuzmin et al., 2009; Rezvani et al., 2010). The nAChR partial agonist varenicline or cytisine that targets α4β2^*^ were found to reduce cue-induced alcohol relapse (Wouda et al., 2011) and the ADE (Sajja and Rahman, 2013a) in animal models. Thus, neurobiological mechanisms associated with relapse are important for new drug developments for alcohol abuse and dependence (McBride et al., 2002; Weiss and Porrino, 2002; Koob and Volkow, 2010).

**Table 2:** **Brain nAChR subtypes and pharmacological agents involved in alcohol/nicotine or substance use disorder**.

**nAChR subtype/addiction disorder**	**nAChR ligand**	**Primary mode of action**
α4β2^*^ AUD or NUD^a^	Cytisine	Partial agonist
α4β2^*^ AUD, NUD, or SUD^b^	Varenicline	Partial agonist
α4β2^*^ AUD, or NUD^c^	Sazetidine A	Desensitizer/partial agonist
α3β4^*^ AUD^d^	CP-601932	Partial agonist
α3β4^*^ AUD^e^	PF-4575180	Partial agonist
α4β2^*^ or other β2 containing subtypes AUD, NUD, or SUD^f^	Mecamylamine	Antagonist
α4β2^*^ or other β2 containing subtypes AUD or SUD^g^	Lobeline	Antagonist
α3β4^*^ NUD^h^	AT-1001	Antagonist
α4β2^*^ NUD^i^	2-fluro-3-(4-nitrophenyl) deschlroepibatidine	Antagonist
α6β2^*^ NUD^j^	α–conotoxin MII	Antagonist

* *Indicates other α or β subunits such as α3, α5, or α6 and β3 or β4*.

*AUD, Alcohol use disorder; NUD, Nicotine use disorder; SUD, Substance use disorder*.

a *Bell et al., 2009; Hendrickson et al., 2009; Chatterjee et al., 2011; Sajja and Rahman, 2011, 2012, 2013a*.

b *Steensland et al., 2007; McKee et al., 2009; Guillem and Peoples, 2010; Hendrickson et al., 2010; Kamens et al., 2010; Bito-Onon et al., 2011; Chatterjee et al., 2011; Wouda et al., 2011; Mitchell et al., 2012; Plebani et al., 2012; Volkow and Skolnick, 2012; Liu et al., 2013; Litten et al., 2013; McKee et al., 2013; Sajja and Rahman, 2013a; Sotomayor-Zarate et al., 2013; Kaminski and Weerts, 2014*.

c *Xiao et al., 2006; Levin et al., 2010; Rezvani et al., 2010*.

d *Chatterjee et al., 2011*.

e *Chatterjee et al., 2011*.

f *Ericson et al., 1998, 2009; Lê et al., 2000; Soderpalm et al., 2000; Blomqvist et al., 2002; Young et al., 2005; Liu et al., 2007; Steensland et al., 2007*.

g *Bell et al., 2009; Hendrickson et al., 2009; Chatterjee et al., 2011; Sajja and Rahman, 2011, 2012, 2013a,b; Roni and Rahman, 2014*.

h *Toll et al., 2012*.

i *Tobey et al., 2012*.

j *Crooks et al., 2014*.

## Nicotinic receptors: targets to treat nicotine or other drug dependence

Evidence utilizing a wide variety of pharmacological and molecular approaches indicates the important role of nAChRs in modulating nicotine self-administration and associated neurochemical effects (Corrigall et al., 1992; Tuesta et al., 2011). As with alcohol abuse and its treatment, nicotinic ligands that target α4β2^*^ subtypes, particularly those expressed in the mesolimbic DA system, show promise for the management of nicotine addiction (Damaj et al., 1997; Coe et al., 2005; Rollema et al., 2007, 2010; Benowitz, 2009). Several therapeutic drug candidates which are either partial agonists or antagonists at nAChRs have been investigated for nicotine taking behavior in preclinical and clinical studies. Varenicline, an FDA approved medication for smoking cessation and an analog of cytisine, is a partial agonist at α4β2-containing nAChRs with higher affinity for this subtype compared to other nAChRs (Coe et al., 2005; Mihalak et al., 2006). Cytisine, a plant alkaloid and a partial agonist at α4β2 nAChRs (Tutka and Zatoñski, 2006), has been tested in various preclinical models associated with nicotine addiction and is approved for smoking cessation in Europe. Sazetidine-A, a novel nAChR desensitizing agent and partial agonist with high selectivity for α4β2 receptors, has been shown to reduce nicotine self-administration in preclinical models (Levin et al., 2010; Rezvani et al., 2010). Bupropion, an FDA approved smoking cessation agent was believed to target α3β2 and/or α4β2 nAChR subtypes in addition to its primary inhibitory mechanisms on the dopamine and norepinephrine transporters (see Crooks et al., 2014). Mecamylamine, a non-selective antagonist at nAChRs, has been investigated and appears to have some efficacy for smoking cessation in a number of clinical studies (Lundahl et al., 2000; Schnoll and Lerman, 2006). Mecamylamine was also reported to decrease cue-induced reinstatement of nicotine-taking behavior (Liu et al., 2007) likely by targeting β2–containing nAChR subtypes. Nevertheless, the clinical efficacy of mecamylamine is tempered by its peripheral side effects (Rose, 2009). Recently, lobeline, a non-selective antagonist at nAChRs, was found to reduce nicotine withdrawal-induced depression-like behavior; again, likely by targeting β2–containing brain nAChRs (Roni and Rahman, 2014). Thus, lobeline may also have potential in preventing smoking relapse by counteracting nicotine withdrawal-induced depression in humans. Similarly, emerging preclinical studies suggest that selective antagonists at α–conotoxin MII (α–CtxMII) sensitive nAChR subtypes have great promise in reducing nicotine self-administration (Crooks et al., 2014).

Early work showed that the novel nAChR antagonist N,N'-dodecane-1,12-diyl-bis-3-picolinium dibromide reduced nicotine self-administration and nicotine-induced DA function and release in reward-relevant brain regions in preclinical models (Neugebauer et al., 2006; Rahman et al., 2007; Dwoskin et al., 2009), suggesting a possible drug candidate for treating nicotine addiction. Recently, 2-fluro-3-(4-nitrophenyl) deschlroepibatidine, a potent α4β2 nAChR antagonist was found to decrease nicotine self-administration in rats (Tobey et al., 2012). In addition, AT-1001, a high-affinity and selective antagonist at α3β4 nAChRs reduced nicotine self-administration in rats (Toll et al., 2012), suggesting an important role for this α3β4 subtype in nicotine addiction. Taken together, these data indicate that nAChRs, specifically, α4β2^*^ α3β2^*^ are important therapeutic targets for all three phases of nicotine addiction, including acquisition and maintenance of nicotine- taking behavior, withdrawal symptoms associated with cessation of nicotine-intake and vulnerability to relapse behavior. Thus, emerging partial agonists and/or antagonists (see Table 2) at nAChRs have therapeutic potential that needs to be further investigated and developed for clinical management of nicotine addiction.

Additional drugs such as galantamine, an acetylcholinesterase (AChE) inhibitor and positive allosteric modulator of α7 and α4β2 nAChRs (Harvey, 1995) has been shown to reduce both nicotine self-administration and reinstatement of nicotine-seeking behavior in animal models (Hopkins et al., 2012). Similarly, rivastigmine, another AChE inhibitor attenuates tobacco craving and smoking in alcohol- and methamphetamine-dependent smokers (Diehl et al., 2009; De La Garza and Yoon, 2011) and desire to use methamphetamine in the latter (De La Garza et al., 2012). Recently, varenicline was found to reduce the positive subjective effects of methamphetamine in human volunteers suggesting a treatment option for methamphetamine dependence (Verrico et al., 2014). This highlights the important role of the nAChR system in poly-drug abuse and dependence. Taken together, these preclinical and clinical studies suggest that AChE inhibitors likely affect nicotine taking behavior by targeting nAChRs and ACh levels along with their modulation of other neurotransmitter systems (Hopkins et al., 2012).

With regard to other psychostimulants, a number of nACR antagonists were found to decrease cocaine self-administration, prevent cue-induced craving for cocaine, and to decrease cocaine effects in a place preference paradigm or reduce cocaine-induced behavioral sensitization (Levin et al., 2000; Zachariou et al., 2001; Champtiaux et al., 2006; Hansen and Mark, 2007) suggesting a direct involvement of nAChRs in cocaine-taking and -seeking behavior. In addition, recent studies indicate that varenicline reduces cocaine-induced reward in rodents and humans (Guillem and Peoples, 2010; Plebani et al., 2012). In contrast, varenicline was found ineffective in reducing cocaine self-administration in a primate model (Gould et al., 2011), indicating mixed effects across models which may be due to species' differences. Consistent with behavioral studies, systemic application of nAChR antagonists significantly reduces cocaine-induced increases in mesolimbic DA-release (Zanetti et al., 2006). Thus, again, both behavioral and neurochemical evidence support an important role for nAChRs in cocaine-taking and -seeking behavior. Similar to its effects on cocaine, nAChRs appear to mediate cannabinoid addiction as well. For example, methyllycaconitine, a α7^*^ nAChR antagonist was found to reduce 9-tetrahydrocannabinol or cannabinoid-1 receptor agonist-induced behavioral and neurochemical effects in animal models, suggesting a critical role in regulating the rewarding effects of cannabinoids (Solinas et al., 2007). Similarly, other animal studies suggest that nAChRs are also important therapeutic targets for treating opiate addiction (Glick et al., 2002; Biala and Staniak, 2010; Hart et al., 2010; Feng et al., 2011). Overall, emerging data indicates that nAChRs are important targets for psychostimulant abuse and addiction, which will probably involve targeting specific nAChR subtypes and their neuromodulatory mechanisms.

## Nicotinic receptor gene variations and addiction

Finally, given the increasingly recognized role of pharmacogenetics/pharmacogenomics in the treatment of addiction (e.g., King et al., 2012; Uhl et al., 2014), it is important to provide a general statement on some of the polymorphisms with a significant association to the initiation, maintenance, relapse, craving and/or treatment outcomes related to addiction. Given the primary addictive component of ingested tobacco is nicotine, it stands to reason that by far the addictive behavior most commonly examined, regarding its association with the nAChR, is nicotine/tobacco addiction. In Indian subjects, variations in the CHRNA5 risk polymorphism (rs16969968) are associated with increased probability of nicotine dependence (Anantharaman et al., 2014). The CHRNA5 risk polymorphism (rs16969968) also has a significant association with nicotine addiction strength (level of physical addiction) with different allelic expression conferring either increased or decreased levels of nicotine dependence (Wojas-Krawczyk et al., 2012). In addition, the CHRNA5 risk polymorphism (rs16969968) is associated with significant increases in fMRI activity of women shown smoking images (Janes et al., 2012). Similarly, in female Canadian citizens of Ontario, the presence of the CHRNA5 risk polymorphism (rs16969968) significantly increased the probability of heavy smoking, whereas the presence of the CHRNA3 polymorphism (rs578775) significantly decreased the probability of heavy smoking (Conlon and Bewick, 2011).

Other work has shown that the normal nicotine metabolizing CYP2A6 genotype can increase the positive association of the CHRNA3 risk polymorphism (rs1051730) with nicotine dependence (Wassenaar et al., 2011). The CHRNA3 risk polymorphism (rs1051730) has been shown to have a significant association with level of nicotine in two heavy smoking regional Italian populations (Sorice et al., 2011). The CHRNA3 risk polymorphism (rs1051730) also has a significant association with short-term (4 week) nicotine abstinence-rates in treatment-seeking smokers (Munafo et al., 2011); although, another study did not find a significant association between this polymorphism and willingness to quit smoking (Marques-Vidal et al., 2011). Two other polymorphisms that have significant predictive value for smoking cessation following treatment with a transdermal nicotine patch and/or bupropion are the CHRNA5 (rs680244) and CHRNB4 (rs12914008) polymorphisms (Sarginson et al., 2011). In an early Finnish study, it was found that variations in the CHRNG-CHRND gene cluster on chromosome 2 were significantly associated with cotinine levels but not number of cigarettes smoked per day suggesting a possible gene-gene interaction with nicotine metabolizing genes (Keskitalo-Vuokko et al., 2011).

Variants in the CHRNA2 and CHRNA6 on chromosome 8 are also associated with increased risk for nicotine dependence in Americans, with the latter being significant in both those of European and African descent and the former being significant in only those of African descent (Wang et al., 2014). A previous study found that the CHRNB3 risk polymorphism (rs1451240) significantly increased the probability of having nicotine dependence in Americans of both European and African descent (Rice et al., 2012). A parallel study found that missense variants in CHRNB4 actually decreased the risk for nicotine dependence in Americans of both European and African descent (Haller et al., 2012). Moreover, these authors reported that an *in vitro* analysis revealed that the minor (protective) allele was associated with increased cellular response to nicotine. In male Japanese subjects, the CHRNB2 polymorphism (rs4845652) may confer protection against nicotine dependence, whereas a combination of this polymorphism with the CHRNA4 risk polymorphism (rs1044397) leads to higher nicotine dependence scores (Chen et al., 2013).

Similar to the study in Japan (Chen et al., 2013), an early study with male Chinese smokers revealed that the CHRNA4 risk polymorphism (rs1044396) was significantly associated with age at smoking initiation and the CHRNA4 risk polymorphisms (rs1044396 and rs1044397) were associated with nicotine dependence (Chu et al., 2011). In subjects from the Center on Antisocial Drug Dependence (CADD), the minor alleles of CHRNA4 risk polymorphisms (rs1044396 and rs1044394) are associated with a significantly greater propensity to develop nicotine dependence than otherwise (Kamens et al., 2013). In Alaska Natives, the nicotine dependence risk polymorphism (rs578776) in the 30 kb CHRNA5-A3-B4 region was significantly associated with level of nicotine intake (Zhu et al., 2013). In European treatment-seeking smokers, the CHRNA4 risk polymorphism (rs3787138) is associated with an increased risk for both nicotine withdrawal and depression (Lazary et al., 2014).

Evaluation of nicotinic receptor gene variations and other addictions include the observation that, in a case-control study on internet addiction in Germany, the CHRNA4 polymorphism rs1044396 occurred significantly more often in those presenting with internet addiction than their controls (Montag et al., 2012). Using a nationally representative sample, significant associations between CHRNA6 polymorphisms (rs1072003, rs2304297, and rs892413) as well as CHRNB3 polymorphism (rs13280604) and excessive alcohol-drinking behavior have been reported (Hoft et al., 2009). In other work, a study from the Nicotine Addiction Genetics consortium in Finland reported a significant association between the CHRNB4 polymorphism rs11636753 and regular alcohol drinking with comorbidity for depression (Broms et al., 2012). Moreover, these authors reported that the effect appeared to be driven primarily by the females in the sample suggesting a sex-dependent effect. In earlier studies than those discussed above it was reported that the CHRNA5 risk polymorphism (rs16969968) is not only associated with nicotine dependence but it is also associated with opioid (Erlich et al., 2010) and cocaine (Sherva et al., 2010) dependence as well. Other CHRNA5 polymorphisms (rs615470 and rs684513) have significant associations with alcohol and cocaine dependence, respectively (Sherva et al., 2010). Another study examining polymorphisms within the CHRNA5-A3-B4 gene cluster found a significant association with the age at initiating drug use across multiple types of drugs of abuse (Lubke et al., 2012). Combined, these findings indicate that multiple polymorphisms associated with nAChR gene have been identified that predict dependence to a number of abuse substances or associated behaviors across national, ethnic and psychiatric groups. The fact that these results span several populations supports the reliability of these findings.

## Summary and conclusions

Due to the limited efficacy of existing FDA approved medications as indicated by continued significant relapse rates, there is a great impetus for determining alternative neuronal brain targets and strategies in the treatment of addiction. As outlined above, significant progress has been made in determining the role that the nicotinic cholinergic system plays in alcohol and drug dependence through both preclinical and clinical studies. Therefore, modulation of brain nAChRs represents a potential therapeutic strategy for treating alcohol and drug dependence. In general, the variety of nAChR subtypes, the respective stoichiometry profile of their respective subunits, their specific localization within the brain, and downstream effects from nAChR activation have been shown to mediate, at least in part, the complex behavioral and neurobiological effects of alcohol and drugs of abuse. Recent studies support the clinical management of alcohol dependence with varenicline and other nAChR partial agonists and/or antagonists, such as mecamylamine, especially among heavy smokers. Further, both chronic alcohol drinking and chronic nicotine exposure affect neural circuits (e.g., hippocampus and prefrontal cortex) mediating cognitive activities such as attention and decision-making. Hence, the use of nAChR-based ligands could improve impaired cognitive function associated with chronic alcohol or nicotine exposure facilitating cognitive and behavioral treatments targeting addiction. Regarding reward and reinforcement, chronic alcohol and drug use enhances cholinergic activity within the mesocorticolimbic dopamine system (e.g., ventral tegmental area) that causes desensitization of nAChR-mediated activity. Therefore, the level and role of neuroplastic changes within this reward system requires further investigation. While the development of nAChR subtype-specific ligands holds great potential for future pharmacotherapies targeting alcohol and drug dependence, possible adverse side-effects associated with these ligands reiterates the need to study these effects before committing them to clinical use. Nevertheless, the substantial health burden that alcohol and drug addiction place on society mandates the recognition that clinical efficacy may outweigh the possible side-effects of a particular nicotinergic system modulator.

In addition, while a global role for the cholinergic system in addiction has been established, the unique role for specific nAChR subtypes has yet to be determined. As these issues are studied, the field will have a better understanding of the neurocircuitry as well as cellular and molecular processes involved in alcohol and drug dependence. With this knowledge, we will be able to develop small molecules that can disrupt, and possibly reverse, the addictive process associated with the cholinergic system's, as well as its control of other neuromodulatory systems, mediation of alcohol and drug dependence. Despite some mixed results, or limited outcomes, of clinical or human laboratory trials using some of these nAChR ligands, there remains considerable potential for additional translational research on the cholinergic system in developing therapeutic management strategies for alcohol and drug dependence. The breadth of these findings in combination with a substantial literature on Genome Wide Association Studies, argue persuasively that future drug development will include small molecules targeting central cholinergic activity resulting in more effective treatments for alcohol, nicotine and other drug addictions.

### Conflict of interest statement

The authors declare that the research was conducted in the absence of any commercial or financial relationships that could be construed as a potential conflict of interest.
